# Age-Related Loss of Cohesion: Causes and Effects

**DOI:** 10.3390/ijms18071578

**Published:** 2017-07-22

**Authors:** Jin-Mei Cheng, Yi-Xun Liu

**Affiliations:** 1 State Key Laboratory of Stem Cell and Reproductive Biology, Institute of Zoology, Chinese Academy of Sciences, Beijing 100101, China; chengjinmeixdg@163.com; 2 Institute of Reproductive Medicine, School of Medicine, Nantong University, Nantong 226001, China

**Keywords:** age, cohesion, aneuploidy, oocytes

## Abstract

Aneuploidy is a leading genetic cause of birth defects and lower implantation rates in humans. Most errors in chromosome number originate from oocytes. Aneuploidy in oocytes increases with advanced maternal age. Recent studies support the hypothesis that cohesion deterioration with advanced maternal age represents a leading cause of age-related aneuploidy. Cohesin generates cohesion, and is established only during the premeiotic S phase of fetal development without any replenishment throughout a female’s period of fertility. Cohesion holds sister chromatids together until meiosis resumes at puberty, and then chromosome segregation requires the release of sister chromatid cohesion from chromosome arms and centromeres at anaphase I and anaphase II, respectively. The time of cohesion cleavage plays an important role in correct chromosome segregation. This review focuses specifically on the causes and effects of age-related cohesion deterioration in female meiosis.

## 1. Introduction

In both males and females, meiosis consists of two rounds of chromosome segregation following a single round of DNA replication, which produces the haploid sperm or egg. Deteriorating effect of the mutant type on sperm characteristics does not impact on embryo development after fertilization in vitro [1]. Male meiosis occurs post-puberty with waves of spermatogenesis that produce mature sperm. Conversely, in females, meiosis initiates during fetal development and has two cell cycle arrests at the germinal vesicle (GV) and metaphase II (MII). This long time interval means that maternal age can affect chromosome separation to produce aneuploidy. Numerous studies have reported that the aneuploid rate in oocytes increases along with maternal age [2,3,4,5,6]. For example, Cheng et al. observed that the incidence of aneuploidy in older mice (31.6%, 12 months) was significantly increased compared with young mice (4.9%, 1 month) [2]. Merriman et al. found that this rate could reach up to 60% in 15 month old mice [3]. In humans, the incidence of aneuploidy increases from 20% in patients who are 35 years of age to over 40% in patients who are 40 years of age [5,6]. Regarding the origin of aneuploidy in oocytes, genetically altered animals and natural aging models both support the hypothesis that the deterioration of cohesion with increasing maternal age represents a leading cause of age-related aneuploidy [7,8,9,10], and this hypothesis has been reviewed in detail by Jessberger [8] and Chiang [10]. Although premature bivalent separation into univalent [11] or altered microtubule–kinetochore interactions [12] during meiosis I has been suggested as the primary defect responsible for age-related aneuploidy, both events have a tight correlation with age-related cohesion deterioration [13,14,15]. In this report, we review recent data on factors that determine the cause of age-related cohesion deterioration and discuss the relationship between cohesion deterioration and some factors associated with chromosome segregation errors.

## 2. Mammalian Cohesion

**Figure 1:**
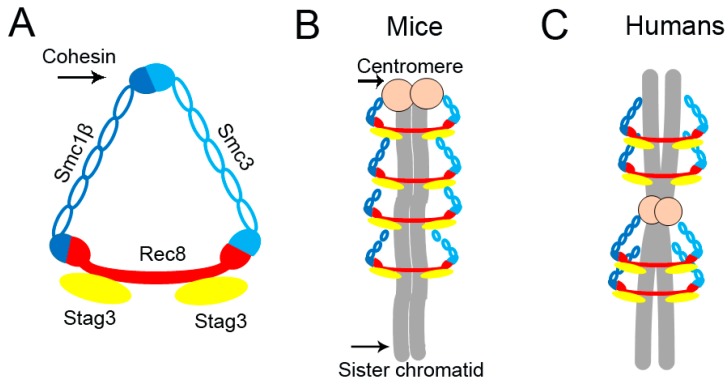
Schematic of cohesin structure in mice and humans. (**A**) The cohesin complex comprises four subunits, Smc1β, Smc3, Stag3 and Rec8, and surrounds sister chromatids in a ring-like protein structure in mice (**B**) and humans (**C**).

Sister chromatids are kept together by the cohesin complex, which generates sister chromatid cohesion [16]. The cohesin complex is a ring-like protein structure wrapped around chromosome centromeres and arms [17,18] (Figure 1B,C). Chromosome segregation requires the release of sister chromatid cohesion from chromosome arms at anaphase I, which permits chiasmata resolution and the segregation of homologous chromosomes. However, centromeric cohesin in meiosis I is protected by Shugoshin until the metaphase II to anaphase II transition, when it is resolved to ensure proper sister chromatid segregation. The timing of cohesin cleavage and where it occurs both play a vital role in correct chromosome segregation.

In mice and humans, the cohesin complex comprises two structural maintenance of chromosomes (Smc) proteins, Smc1β and Smc3, which heterodimerize to form a large V-shaped structure [19,20,21]. The open side of the cohesin complex is closed by a member of the kleisin protein family, Rec8, which ultimately forms a tripartite ring [22,23,24,25]. A fourth protein, Stag3, associates with the kleisin and forms the complex [22,26,27] (Figure 1A).

Kleisin Rec8 has an essential role in mammalian meiosis in that Rec8-null mice of both sexes have germ cell failure and are sterile [28]. Rec8 is a meiosis-specific cohesin protein [24,29] and it appears shortly before the premeiotic S phase in the nucleus [24,30]. Rec8 is cleaved by separase, which is necessary for chromosome segregation [31]. In oocytes from old mice, chromosome-associated Rec8 is severely reduced, which is a leading cause of chromosome segregation errors [32,33]. Chromosome segregation must be accurate because the Rec8 variant is not substantially reduced during female fertility [31]. In other words, a threshold level of Rec8 is necessary to prevent errors, which may be ~10% or less than the starting amount, and only after chromosome-associated Rec8 reaches this threshold does the number of chromosome segregation errors increase [32]. On the other hand, Tachibana-Konwalski et al. [34] demonstrated that Rec8-containing cohesin maintained bivalents without turnover during the growth phase of mouse oocytes. In 2016, Burkhardt et al. found that Rec8-Myc was capable of establishing functional cohesion when it was activated before meiosis, but that it cannot be activated after meiotic DNA replication in fetal oocytes [35]. In mouse female meiosis, they posited the hypothesis that the inability of oocytes to build cohesion during the dictyate arrest that lasts for months or decades contributes to the maternal age-related chromosome missegregation and the production of aneuploidy fetuses [35]. In addition, cohesin subunit Smc1β has also been reported to be expressed only during prophase I prior to the primordial follicle stage to ensure sister chromatid cohesion until mice reach an advanced age [9,36]. Therefore, the inability of oocytes to reload cohesion on a chromosome during prolonged prophase arrest is one of the important causes of age-related chromosome segregation errors. Although new cohesive linkages that are established after meiotic S phase by Nipped B and the acetyltransferase Eco in *Drosophila* oocytes have been demonstrated [37], the rejuvenation program can no longer supply new cohesive linkages at the same rate at which they are lost during maternal aging [34,37].

## 3. The Cause of Age-Related Loss of Cohesion

### 3.1. Increased Sensibility of Separase 

**Figure 2:**
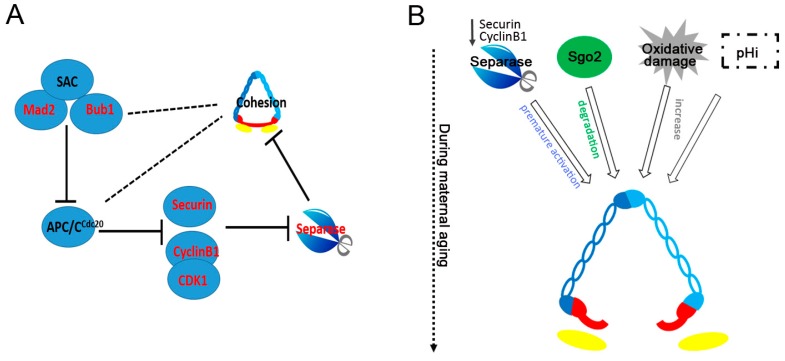
The cohesin-cleaving factors during maternal aging. (**A**) The correlation between spindle assembly checkpoint (SAC) and anaphase-promoting complex or cyclosome in association with Cdc20 (APC/C^cdc20^) and cohesion. The active SAC causes APC/C^Cdc20^ inactivation, which cannot degrade securin and cyclin B1 to cause the cleavage of cohesion structure and anaphase onset. Therefore, the SAC and APC/C^cdc20^ indirectly regulate the cohesion structure (indicated by broken line). Full line T shows the inhibitive effects of two factors; (**B**) Premature activation of separase, Sgo2 degradation, and the increase of oxidative damage and intracellular pH may be the leading cause of cohesion-ring structure cleavage during maternal aging.

Anaphase is triggered when ring-shaped cohesion is cleaved by separase, which is a large cysteine endopeptidase that cleaves the kleisin subunit of cohesin (Scc1/Rad21 in mitosis, Rec8 in meiosis) [38,39,40]. Before anaphase, the activity of separase is inhibited by two independent mechanisms: Binding to securin and inhibitory phosphorylation by cyclin-dependent kinase Cdk1 [41,42]. Cdk1 stably binds to phosphorylated separase via its regulatory cyclin B1 subunit [43]. The AA-separase mutant (AA-separase) is constructed by mutating both CDK1 phosphorylation sites, including S1121A and T1342A [42]. Depletion of securin is induced through the injection of morpholino (MO) antisense oligonucleotides [44]. When the cytoplasm of germinal vesicle-intact oocytes are microinjected with securin MO, AA-separase cRNA, or AA-separase cRNA and securin MO together (AA + MO), all chromosomes in meiosis I are intact in uninjected, AA-separase, and securin MO oocytes, whereas the majority of AA + MO oocytes show premature separation of both bivalents and sister chromatids [45]. Therefore, both mechanisms must be disrupted to prematurely activate separase. In addition, lower concentrations of both AA-separase cRNA and securin MO can significantly cause more old oocytes with prematurely separated bivalents and sister chromatids compared with young oocytes [45]. Specifically, cohesin can be removed by the low activity of separase in aged mouse oocytes. Given that the proteolytic activity of separase is essential for Rec8’s removal from chromosome arms [40], it may be possible that leaky separase activation starts after birth and lasts until old age or low levels of separase activity could cause age-related loss of cohesion (Figure 2B).

### 3.2. The SAC and APC/C^Cdc20^

The loss of cohesion around chromosome arm at the onset of anaphase in mouse oocytes depends on proteolysis of the separase inhibitor securin and the Cdk1 regulatory subunit cyclin B1 [41], which is caused by a ubiquitin protein ligase called the anaphase-promoting complex or cyclosome (APC/C) in association with Cdc20 (APC/C^Cdc20^) [46,47]. Without cyclin B1 and securin degradation, cell cycle progression is halted because CDK1 activity remains high and chromosome cohesion is maintained. In other words, APC/C^Cdc20^ indirectly triggers the loss of sister-chromatid cohesion (Figure 2A). However, APC/C must not be activated until chromosome bi-orientation has been completed. The regulatory mechanism responsible for delaying anaphase in this manner is known as the spindle assembly checkpoint (SAC) [48,49]. The checkpoint protein Mad2 catalyzes the sequestration of the APC/C^Cdc20^ activator protein to block its ability to ubiquitinylate either securin or cyclin B by the Cdc20/Mad2 complex binding to the APC/C when unattached, mono-oriented, or syntelically attached kinetochores exist [50,51]. Depletion of Mad2 through the injection of morpholino antisense oligonucleotides results in aneuploidy and advances the onset of cyclin B and securin destruction [52]. Mad2 overexpression inhibits homolog disjunction [52,53]. Bub1 is a key SAC component, and its loss in oocytes causes precocious loss of cohesion between sister centromeres and massive chromosome missegregation at meiosis I [49], which has also been reported in other cells [54]. Centromeres in Bub1-deficient cells also separate prematurely. However, this separation is a consequence of SAC dysfunction rather than a direct role for Bub1 in protecting centromeric cohesion [54]. Therefore, it is possible that SAC has an important role in the maintenance of cohesion by controlling APC/C^Cdc20^ activity to operate effective separase activation in oocytes (Figure 2A). In addition, in yeast, Bub1 is necessary for the recruitment to centromeres of Sgo1 that protects cohesin from separase during meiosis I [55,56]. Bub1 might act in a similar fashion in oocytes and help to recruit Sgo1 or Sgo2 to centromeres.

In human and mouse oocytes, aging causes a reduction in Mad2 and Bub1 at the transcriptional level [57,58,59]. Mad2 on kinetochores had a significant ~30% reduction in oocytes of aged mice at 3 h after resumption of meiosis I. This reduction was apparent even at 5–7 h after germinal vesicle breakdown after a 10 µM nocodazole treatment [60]. Furthermore, the kinetochore localization of both Bub1 and Bubr1 proteins decreases with age in human oocytes [61]. A recent study indicates that Smc1β is essential for activation of SAC activity during mouse oocyte meiosis [62]. Therefore, there might be some association between decreased kinetochore localization of the SAC proteins and cohesion loss with maternal age, including the possibility that decreased SAC ability in aged oocytes promotes the APC/C^Cdc20^ to degrade cyclin B1 and securin, which causes the loss of sister chromatid cohesion [63] (Figure 2A).

### 3.3. Premature Shugoshin 2 Degradation 

Two shugoshin isoforms have been identified to protect centromeric cohesin in mammals, specifically Sgo1 and Sgo2 [56,64,65]. Both isoforms are expressed in mouse germ cells, and Sgo1-depleted oocytes similarly exhibit largely intact cohesion of the sister chromatids. By striking contrast, however, separated single chromatids were prevalent in Sgo2-depleted mouse oocytes [64,66]. Sgo2 distribution in human oocytes could not be described due to the lack of a working antibody, but mechanisms to protect centromeric cohesion in meiosis are essentially conserved across eukaryotes, including mammals. Therefore, Sgo2 alone plays a predominant role in protecting centromeric cohesion in meiosis I in oocytes, whereas Sgo1 is not required for this function [64,66].

In meiosis I, Sgo2 cooperates with protein phosphatase 2A (PP2A) to protect Rec8 at centromeres from cleavage by separase [64,67,68]. Any premature Sgo2 loss in meiosis I would make centromeric cohesin vulnerable to separase-mediated cleavage, which generates single chromatids and has been observed in aged mice [7,69]. The Sgo2 signal on chromosomes in oocytes from 14-month-old mice was obviously reduced by immunostaining [7]. Furthermore, the phenomenon that the largest sister interkinetochore distance tended to have reduced Sgo2 in aged MII oocytes also shows a strong association between an age-related loss in sister chromatid cohesion and reduced Sgo2 [69]. Interestingly, *Smc1β^—/—^* oocytes have reduced levels of chromosome-associated Sgo2 [7], which indicates that the recruitment or retention of Sgo2 is influenced either directly or indirectly by the level of cohesin in mouse oocytes. This phenomenon was also found in maize meiosis [70]. Therefore, reduced levels of chromosome-associated Sgo2 during oocyte aging can give rise to a loss of cohesin, which in turn may amplify loss of Sgo2 (Figure 2B).

Waplin (Wapl) is a cohesin-binding protein that promotes sister-chromatid resolution in mitotic prophase [71,72]. Sororin, which is a substrate of the anaphase-promoting complex, is required for sister chromatid cohesion in vertebrates [73,74,75]. In human somatic cells, Sgo1 physically shields cohesin from Wapl in addition to recruiting PP2A to dephosphorylate cohesin and sororin [76]. Direct antagonism between Sgo1 and Wapl augments centromeric cohesion protection [76]. This mechanism, or a similar one whether also existing in meiosis remains unknown.

### 3.4. Oxidative Damage

Oxidative damage increases with age in different organisms and cell types [77,78,79]. In aging cells, the increase in oxidative damage is caused, in large part, by reactive oxygen species (ROS) [80]. The accumulation of oxidative damage over a lifetime negatively impacts oocyte quality, including meiotic division and chromosomal segregation [78,81,82]. Antioxidant therapy positively counteracts the bad effects of maternal aging on chromosome segregation in mouse oocytes [83]. How does the oxidative damage influence chromosomal segregation in aged oocytes? Perkins et al. [84] tested the hypothesis that increased oxidative damage in older oocytes may be one of the factors that leads to premature loss of cohesion through knockdown of scavenger superoxide dismutase in the *Drosophila* oocyte. Their results indicate that oxidative damage moderately increases the percentage of oocytes with arm cohesion defects and supports the model that accelerates loss of cohesion in aging human oocytes that is caused, at least in part, by oxidative damage (Figure 2B).

### 3.5. Other Factors

Our lab shows that aged oocytes have a high intracellular pH value (pHi) and loss of cohesion occurs when young oocytes have a high pHi. Thus, we inferred that dysregulation of pHi in aged oocytes might damage protein–protein binding affinity or protein localization of cohesion subunits, which leads to deterioration of chromosome cohesion [2] (Figure 2B), but the specific mechanism remains unknown. Other factors might also contribute to cohesion deterioration, such as spontaneous hydrolysis of peptide bonds, cohesion deacetylases and releasins. Cleavage or spontaneous hydrolysis of a single peptide bond at any site within the large cohesin ring could open the tripartite ring and eliminate the topological linkage of two sister chromatids [8]. Loss of histone deacetylase 8 (HDAC8) activity results in increased SMC3 acetylation and inefficient dissolution of the ‘used’ cohesin complex released from chromatin in both prophase and anaphase of mitosis, which suggests that histone deacetylase may have a role in diminishing centrosome cohesion from chromosomes [85,86]. In mitosis, cohesion loading onto chromosomes is mediated by the entry of DNA into cohesion rings, whereas dissociation is mediated by facilitating the exit of DNA via a transiently open Smc3–kleisin interface, which is a process regulated by Smc3 acetylation [87]. However, whether these mechanisms have similar functions in oocyte meiosis or contribute to the loss of cohesion during maternal aging has yet to be demonstrated.

## 4. The Effects of Age-Related Loss of Cohesion

### 4.1. Destabilization of Chiasmata

**Figure 3:**
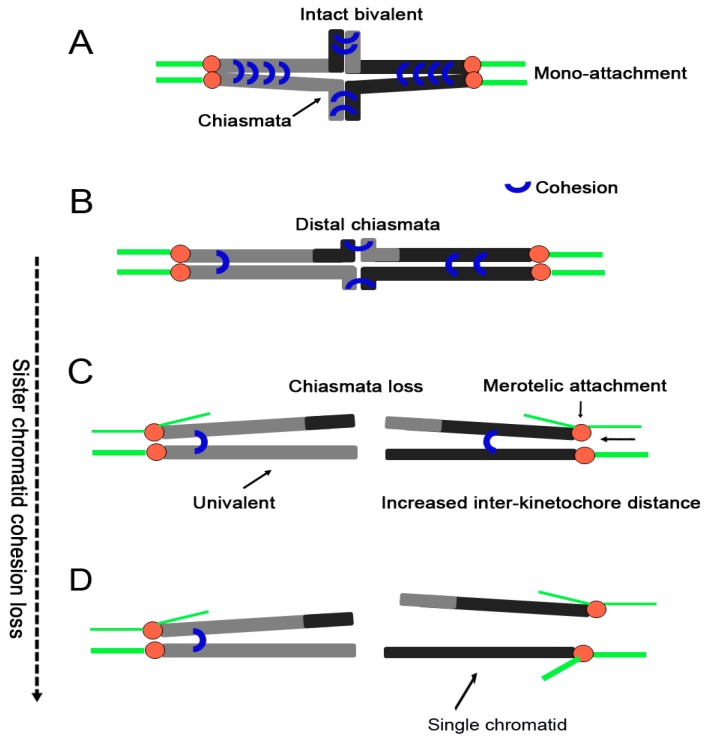
The effects of age-related cohesion loss. (**A**) An intact bivalent configuration is attached by an amphitelic microtubule bundle. The two sister chromatids of each homologous chromosome face towards the same spindle pole. The cohesion embraces the two sister chromatids at their centromeres and along the chromosome arm; (**B**) The chiasmata shift toward the distal chromosome with cohesion loss; (**C**) A bivalent configuration becomes two univalents at the time of chiasmata loss, which gives rise to arm cohesion deterioration; (**D**) Centromere cohesion loss can generate a single chromatid in oocytes. Centromere cohesion loss can cause an increase in sister inter-kinetochore distance, as well as the merotelic attachment of a sister kinetochore and the appearance of a single chromatid. Red circle, centromere; green line, microtubule.

Chiasmata have long been recognized as a regular feature of meiosis and are the result of prior crossing over between homologous chromatids [88]. They are formed during fetal development and are not resolved until shortly before ovulation occurs in a sexually mature female. Segregation of homologous chromosomes during meiosis depends on chiasmata and sister chromatid cohesion at anaphase I [89] (Figure 3A). What is the relationship between chiasmata and cohesion? Bickel et al. demonstrated that *Drosophila* oocytes required sister-chromatid cohesion to maintain chiasmata between recombinant chromosomes [90]. In mammalian oocytes, it has been found that the cohesin Smc1β subunit acts as a chiasmata binder to stabilize sites of exchange until anaphase [9]. Rec8 cleavage triggers chiasmata resolution during mouse meiosis I [34]. Thus, sister chromatid cohesion is required to stabilize chiasmata. In support of this conclusion, it was reported that this cohesion can also keep chiasma in place during meiosis I [88].

Distally associated homologs without a visible chiasmata are prevalent in 14 month old mouse oocytes [7]. Meanwhile, reduced cohesion on chromosome arms is accompanied by an increase in distal chiasmata in old mouse oocytes ([32], detailed in the supplementary material), which is consistent with the idea that there is a shift toward distal chiasmata in oocytes from aged mice [91] (Figure 3B). In other words, a defect in cohesion distal to crossover sites results in a shift of chiasmata placement (chiasmata slippage; Figure 3B) or even premature bivalent separation in mouse meiosis I (Figure 3C). Thus, destabilization of chiasmata in association with cohesin depletion is a general feature of mouse aging. In addition, bivalents, whose integrity is compromised by destabilization of chiasmata [88,92], would be unable to establish the tension required for stable biorientation of homologs.

In humans, the placement of crossover (chiasmata) sites varies according to gender. In females, chromosomes tend to have more crossover sites than males, and longer chromosomes form more crossovers than shorter ones [93,94]. When crossover sites form close to telomeres, reduced amounts of cohesin may link homologous chromosomes, whereas crossover sites within centromeres may compromise sister chromatid cohesion or prevent the removal of cohesins, which causes nondisjunction errors [95,96]. Researchers have proposed that a minimal amount of the sister chromatid cohesion complex remaining distal to the exchange event is expected to increase the risk of meiosis I errors [96,97].

### 4.2. Bivalents Separation into Univalents

The configuration of two linked homologous chromosomes is called bivalent (Figure 3A). The integrity of bivalents is crucial for accurate chromosome segregation. Weakly attached bivalents are common and some bivalents can even split into individual chromosomes, called univalent, because of cohesion loss in aged human and mouse oocytes [11,13,69]. Sakakibara et al. found that bivalent separation precedes the majority of segregation errors during meiosis I in aged mouse oocytes, and they also determined that univalents are predisposed to predivision in human oocytes through chromosome tracking [11]. The prevalence of univalents increases dramatically with age and occurs in 40% of oocytes from women older than 35 compared with 10% of oocytes from women who are 30–35 years of age [13]. Age-related cohesion loss within bivalents is not restricted to centromeric regions. Cohesion linking of homologous chromosomes is also compromised. For example, *Smc1β*-knockout oocytes that have a reduced amount of cohesin exhibit an age-related increase in univalents [9]. Tobacco etch virus-induced Rec8 cleavage triggers chiasmata resolution and converts bivalents to univalents [34]. Separated sister kinetochores allow bivalents to rotate and twist by 90 degrees on the spindle in aged human oocytes [13]. These studies all indicate that age-related cohesion loss can cause bivalent configurations to separate into univalent configurations (Figure 3C). The fate of these univalents may be to bi-orientate and so divide equationally in metaphase I (MI) [15]. Alternatively, they may divide intact during MI and form interactions with microtubules that satisfy the SAC without bi-orientation [15], which is similar to the results for some univalents from the XO mice that have been reported [98]. However, both situations may cause chromosome segregation errors and aneuploidy. Thus, how to reduce this phenomenon is an outstanding problem that we should attempt to solve in the future.

### 4.3. Kinetochore Orientation Alteration

Chromosomes must establish stable biorientation prior to anaphase to achieve faithful segregation during cell division [99]. In meiosis I, sister chromatids become attached from the same pole and co-segregate, and homologous chromosomes connected by chiasmata segregate to opposite poles. In meiosis II, sister chromatids become attached at the kinetochore by spindle microtubules emanating from opposite poles and are segregated equationally. There are stochastic interactions between microtubules and kinetochores through the “search and capture” mechanism [100,101]. Therefore, two-thirds of all biorientation attempts were erroneous and that 86% of all homologous chromosomes required error corrections of their kinetochore microtubule attachments before they established a stable biorientation in mouse oocytes [99].

When a single kinetochore binds microtubules that are oriented toward both spindle poles, a prominent error called merotely will occur [102,103,104]. Researchers indicate that the mitotic spindle checkpoint cannot detect this attachment orientation, which can induce chromosome lag during anaphase [103,105]. Consistent with mitotic anaphase, merotelic attachment persists during meiotic anaphase II and could cause chromosome lag and tailing [106,107]. Tension established through merotelic attachment of chromatids can maintain spindle checkpoint inactivation, which promotes chromatid transmission and the formation of aneuploid cells [103,107]. In mitotic and cancer cells, merotelically attached kinetochores are a major mechanism of aneuploidy and chromosomal instability [103,108]. In old mouse oocytes, this orientation has a significant increase and has been introduced as a lesion to explain aneuploidy in meiosis I and meiosis II [12,106]. Therefore, merotelic attachment may be one of the leading factors to cause age-related aneuploidy.

Cohesion-mediated association of homologous chromosomes promotes proper orientation and microtubule attachments in the meiosis I spindle [100]. Because cohesion makes sister kinetochores behave as a single kinetochore unit during meiosis I, sister kinetochores tend to attach to microtubules in a syntelic manner to make sure that the bivalent configuration is captured from opposite poles and sister kinetochores of each bivalent face towards the same spindle pole. When cohesion is lost, the tension balance of bivalent is broken. For evading surveillance mechanisms that monitor tension, new tension will come from merotelic kinetochore attachment. Furthermore, the cohesin subunit Rec8 has been reported to play an important role in the mono-orientation of sister kinetochores [24]. Sister chromatids are aligned at the metaphase equator by tension balance between poleward pulling forces and sister chromatid cohesion during meiosis II [100]. In other words, cohesion produces tension by counteracting the pulling force of microtubules that capture kinetochores from opposite poles. Therefore, this tension has a crucial role in stabilizing monopolar attachment of kinetochores. In cells, weakened centromeric cohesion has been reported to trigger merotelic attachment [103,109]. In addition, subtle defects in cohesion can disrupt centromere geometry by altering the normal back-to-back configuration of sister kinetochores to increase the formation rate of merotelic attachments [110]. In brief, we speculate that weakened cohesion plays an important role in the increase of merotelic kinetochore attachment in aged oocytes (Figure 3C).

In human oocytes, having separated sister kinetochores increases the risk of merotelic kinetochore-microtubule attachments [13] (Figure 3C). The distance between kinetochores reflects differences in cohesion holding the centromeres together. Increased interkinetochore distances have been found in old mouse and human oocytes [3,32,111]. In mouse oocytes, the distance is 0.25 µm at 3 months old, but reaches 0.38 µm by 12 months old. In 15 month old mice, the average distance even reaches 0.82 µm [3]. In human oocytes, the average interkinetochore distance increases from 0.82 ± 0.03 µm in the youngest females (16.4 years) to 1.1 ± 0.03 µm in the oldest humans (37.3 years) [111]. When the distance between kinetochores is long enough, a single chromatid would appear, which indicates complete loss of centromere cohesion (Figure 3D). This event is called precocious separation of sister chromatids, which is the main defect found in older mice during MII arrest [69].

## 5. Conclusions and Future Perspectives

Experiments in mouse and human oocytes have shown that cohesion loss increases with maternal age, which is a major mechanism of age-related aneuploidy [7,32,33,111]. In this review, we explain how cohesion deterioration in aged oocytes occurs due to the premature loss of Sgo2 protein, the increased sensibility of separase, the SAC and APC/C^Cdc20^, and oxidative damage (Figure 2). These factors alone or together may influence the strength of cohesion with advanced maternal age. However, how to avoid these factors as female age remains unknown. On the other hand, we also discuss the effects of cohesion deterioration on chiasmata stabilization, bivalent integrity, kinetochore orientation and sister kinetochore distance (Figure 3), which all have a direct connection to chromosome segregation. A greater understanding of the causes and effects of cohesion deterioration during maternal aging may provide opportunities to counteract age-related deterioration of chromosome structures. Caloric restriction (CR) does not reveal age-related increase in oocyte aneuploidy or chromosomal misalignment in rodents [112], and improves postnatal survival of offspring delivered by older females [113]. The percentages of aneuploidy and diploidy significantly decrease in females fed an antioxidant (the mixture of vitamin C and vitamin E) diet comparing with those in control [83]. In addition, it has been reported that the antioxidant melatonin can prevent postovulatory oocyte aging in pigs [114] and mice [115]. The catalase can protect chromosomes from oxidative damages during meiotic maturation in mouse oocytes [116]. Given that age-related cohesion loss is a leading cause of the increase in aneuploidy with advanced maternal age, CR and antioxidants may have a positive effect on maintaining sister chromatid cohesion during maternal aging. Despite the beneficial effect of oral antioxidant administration and CR on oocyte quality, transference of antioxidants and CR to human beings should be made with caution because many undesirable systemic disorders may induce reproductive disturbances, such as pharmacological doses of vitamins C and E. How to maintain oocyte quality and reduce age-related cohesion deterioration during maternal aging are still great challenges for researchers.
